# Advances in Orthopedic Surgery Irrigation: A Review of Traditional Agents and the Emergence of Citrate-Based Solutions

**DOI:** 10.3390/jcm14113681

**Published:** 2025-05-24

**Authors:** Mitchell K. Ng, Afshin E. Razi

**Affiliations:** Department of Orthopaedic Surgery, Maimonides Medical Center, Brooklyn, NY 11219, USA

**Keywords:** intraoperative irrigation, prosthetic joint infections, surgical site infection, citrate-base solutions, biofilm disruption

## Abstract

Surgical site infections (SSIs) and prosthetic joint infections (PJIs) remain significant challenges in orthopedic surgery, particularly in joint arthroplasty. Intraoperative irrigation is a widely used strategy for infection prevention, yet traditional solutions such as saline, povidone-iodine, hydrogen peroxide, and sodium hypochlorite are limited by cytotoxicity, short antimicrobial duration, and poor biofilm penetration. This review evaluates commonly used irrigation agents and highlights the growing evidence supporting a novel citrate-based solution as a potentially superior alternative. These agents combine broad-spectrum antimicrobial activity, effective biofilm disruption through ionic chelation, and prolonged postoperative protection with minimal harm to host tissues. Early clinical studies and ongoing randomized trials have demonstrated promising results, including reductions in postoperative swelling, opioid use, and infection rates. As more data become available, citrate-based solutions may emerge as the preferred standard for intraoperative irrigation in orthopedic procedures.

## 1. Introduction

Surgical site infections (SSIs), bacterial biofilm development, and periprosthetic joint infections (PJIs) are significant complications that undermine surgical success and patient recovery in orthopedic surgery [1,2]. The increasing number of orthopedic procedures—ranging from total joint arthroplasties (TJAs) and spinal fusions to fracture repairs and tumor resections—has correlated with a heightened concern for postoperative infections, particularly in cases involving implanted hardware [3]. Although PJIs occur in less than 2% of patients undergoing joint arthroplasty, the severity of these infections is well documented, with outcomes often compared to those of high-morbidity diseases due to their challenging management and recurrent nature [4]. The economic burden of managing PJIs in the United States alone is projected to exceed $1.85 billion annually by 2030 [5].

A key factor in the pathogenesis and persistence of these infections is the formation of bacterial biofilms on surgical implants and tissues. In the presence of implants or avascular wounds, infections can be triggered by as few as 10 bacterial organisms, compared to the 10,000 to 1,000,000 required in native tissue [6]. While the incidence of implant-related infections remains low ~1–2%, biofilm formation is nearly universal in cases of chronic infection, late-onset infection, and “culture-negative” infection [6]. This highlights the vulnerability of surgical hardware to colonization and the importance of effective intraoperative decontamination [7]. Biofilms confer protection to bacterial colonies, allowing them to evade immune responses and exhibit resistance to antibiotics. This biofilm-mediated defense presents a formidable challenge to surgeons, as standard antimicrobial therapies and mechanical debridement alone are often insufficient to eradicate entrenched bacterial communities [8,9]. Intraoperative irrigation, a long-standing component of perioperative infection control protocols, has traditionally relied on agents such as saline, povidone-iodine, or chlorhexidine [7]. While these solutions provide some antimicrobial activity or mechanical cleansing, they are often limited by brief efficacy, inability to penetrate biofilms, or toxicity to host tissues.

These shortcomings underscore the need for innovative irrigation technologies that are both effective in disrupting biofilms and safe for use on delicate musculoskeletal structures. A novel citrate-based irrigation solution has emerged as a compelling candidate [10,11]. Formulated with citric acid, sodium citrate, and sodium lauryl sulfate, this solution is designed to chelate metal ions essential to the biofilm matrix, break down bacterial adhesion, and emulsify debris from surgical sites [10,12]. The citric components destabilize the extracellular matrix, while the surfactant enhances penetration and debris removal, providing comprehensive wound cleansing without the need for rinsing.

In contrast to traditional agents, the citrate-based solution offers extended antimicrobial activity, reduced toxicity to host tissues such as osteoblasts and fibroblasts, and improved wound healing environments [10]. Early evidence suggests additional benefits such as decreased postoperative swelling, enhanced range of motion, and shorter opioid use duration in joint arthroplasty patients [10,13]. These findings have prompted broader interest in its application beyond arthroplasty, including in spine surgery, trauma, and oncologic procedures, particularly among high-risk patient populations.

This aims of this review are as follows: (1) to explore the historical and current use of irrigation in orthopedic surgery (normal saline, povidone-iodine, hydrogen peroxide, acetic acid, sodium hypochlorite, antibiotic irrigation solutions, and a novel citrate-based irrigation solution); (2) evaluate the comparative efficacy and safety profiles of commonly used agents; and (3) present the scientific rationale for broader clinical adoption of citrate-based irrigation solutions as a preferred adjunct in infection prevention strategies across orthopedic subspecialties.

## 2. Overview of Commonly Used Irrigation Systems in Orthopedic Surgery

Irrigation is an integral part of surgical wound management in orthopedics, used to reduce microbial contamination, remove necrotic tissue and debris, and prepare the wound bed for closure or implantation [7]. A wide variety of irrigation systems are in use, differing in delivery method, volume applied, and type of solution. Each of these variables has implications for the efficacy of bacterial clearance and tissue preservation.

### Delivery Methods

Common irrigation delivery methods include low-pressure irrigation, high-pressure pulse lavage, continuous flow systems, and tangential hydrosurgery. According to the American College of Surgeons, irrigation delivery systems are generally categorized by pressure: low pressure (1–15 psi or 6.8–103 kPa) and high pressure (15–35 psi or 103–241 kPa) [14]. Low-pressure irrigation, typically delivered via gravity-fed systems or bulb syringe, is the standard in primary joint arthroplasty due to its reduced tissue trauma and contamination spread [7]. High-pressure pulse lavage, on the other hand, is frequently utilized in revision or trauma settings because of its superior mechanical debridement [15]. However, it may cause deeper penetration of contaminants into tissues and lead to soft tissue or bone damage [15]. Continuous flow systems are widely used in arthroscopy, providing a clean surgical field but offering limited antimicrobial benefit [16]. Tangential hydrosurgery, a newer modality, uses high-velocity fluid jets to debride wounds with precision, but remains under investigation for widespread orthopedic application [17].

The volume of irrigation used during surgery is influenced by the procedure type and wound status. In clean, primary procedures such as total knee or hip arthroplasty, surgeons typically use 1 to 3 L of irrigation fluid [18]. In contrast, for contaminated wounds or revision surgeries, irrigation volumes can exceed 6 to 9 L [19]. While larger volumes can enhance mechanical cleansing, there is no universally accepted optimal volume, and excessive irrigation may cause fluid overload in soft tissues.

A range of irrigation solutions are employed in clinical practice [7]. Normal saline is the most common due to its low cost and excellent tissue compatibility, although it lacks antimicrobial activity. Povidone-iodine, especially in diluted forms (~0.35%), has been recommended by international guidelines for its broad-spectrum antimicrobial properties [20,21]. However, its cytotoxic effects at higher concentrations and rapid inactivation by blood are limitations. Chlorhexidine gluconate is another widely used agent that exhibits broad antimicrobial activity, but its cytotoxicity and poor penetration into biofilms restrict its utility. Hydrogen peroxide and acetic acid are also employed in certain settings for their antimicrobial effects, but both carry significant risks of tissue damage and are not used routinely [22].

Antibiotic irrigation solutions containing agents like bacitracin or polymyxin B have historically been used, particularly in revision or trauma surgery [23]. Recent evidence and international guidelines, however, caution against this practice due to limited efficacy, risk of hypersensitivity, and concerns about antimicrobial resistance [24]. Finally, citrate-based irrigation solutions have emerged as a novel option. Composed of citric acid, sodium citrate, and sodium lauryl sulfate, this formulation offers a unique mechanism of biofilm disruption while maintaining low cytotoxicity and prolonged antimicrobial effect [10,11]. Unlike other agents, it does not require rinsing after application and may support improved wound healing outcomes by preserving host tissue integrity.

This variety of delivery systems, volumes, and irrigants reflects the diverse needs across orthopedic procedures and highlights the importance of selecting the most appropriate irrigation strategy based on individual surgical scenarios and their effects against organisms (bacteria, mycobacteria, fungi, and spores) and against biofilm (Table 1). Over the past several decades, irrigation strategies in orthopedic surgery have evolved based on surgical complexity and infection risk, with specific solutions and delivery methods tailored for primary orthopedic cases (e.g., primary total knee/hip arthroplasty), revision procedures (two-stage revision for periprosthetic joint infection), trauma (e.g., open fractures), infected wounds (surgical site infections), and musculoskeletal tumor resections.

**Table 1:** Overview of commonly used irrigation systems in orthopedic surgery.

Irrigation Solution	Mechanism of Action	Bacteria	Myco Bacteria	Fungi	Spores	Biofilm Effect
Normal Saline	Mechanical flushing and dilution; no antimicrobial properties	None	None	None	None	None
Povidone-Iodine (Betadine)	Oxidizes cellular components via free iodine; disrupts proteins and fatty acids	Bactericidal	Myco bactericidal	Fungicidal	Sporicidal	Limited effect
Hydrogen Peroxide	Produces reactive oxygen species, damaging proteins and DNA	Bactericidal (G+ > G−)	–	Fungicidal	Sporostatic	Limited effect
Acetic Acid	Lowers pH, denatures proteins and enzymes, increases cell wall permeability	Bactericidal	–	Fungicidal	–	Some effect
Sodium Hypochlorite	Oxidative destruction of cellular proteins and membrane structures	Bactericidal	–	–	Sporicidal (at high levels)	No effect
Antibiotic Irrigation	Targets bacterial replication or protein synthesis depending on antibiotic used	Variable	Variable	None	None	Minimal effect
Citrate-Based Solution	Chelates ions disrupting biofilm EPS; emulsifies debris via surfactant action	Bactericidal	Unknown	Fungistatic	Unknown	High effect

Each irrigation solution has its own advantages/disadvantages, which are detailed below (Table 2).

## 3. Irrigation Types

### 3.1. Normal Saline

Normal saline (0.9% sodium chloride) remains the most commonly used irrigation solution in orthopedic surgery due to its safety, accessibility, and mechanical cleansing capabilities [7]. Composed of isotonic sodium chloride in water, normal saline is physiologically compatible with host tissues and does not induce cellular toxicity, making it an ideal baseline irrigant for clean surgical wounds and as a delivery medium for other agents [25].

The primary mechanism of action of normal saline is mechanical dilution and physical removal of contaminants. It does not possess intrinsic antimicrobial, antifungal, or antiviral activity, nor does it affect biofilm structure or stability [19,26]. Its use is grounded in the principle that “the solution to pollution is dilution”, aiming to decrease microbial burden by volume displacement rather than chemical eradication. Normal saline does not disrupt bacterial cell walls or interfere with biofilm integrity, and it lacks any activity against mycobacteria, spores, or fungal organisms [7].

Normal saline can be delivered through various modalities including bulb syringe, gravity-assisted flow, and pulse lavage [15,19]. In orthopedic surgery, delivery method and volume vary depending on wound type, contamination level, and procedural context. For elective clean cases, volumes of 500 mL to 3 L are commonly used [18]. In contaminated or traumatic wounds, volumes may increase to 6 to 9 L, especially in conjunction with low-pressure delivery systems. The Fluid Lavage of Open Wounds (FLOW) trial, the largest randomized study of irrigation practices to date, found no difference in reoperation rates between high- and low-pressure saline lavage, further supporting low-pressure delivery as a standard approach due to reduced tissue trauma [16].

Despite its lack of antimicrobial properties, normal saline remains a staple in surgical irrigation due to its favorable biocompatibility and low cost. However, in procedures with a high risk of infection—particularly those involving implants or hardware—saline is often supplemented or replaced by antiseptic or biofilm-targeting agents. Its use as a standalone solution may be insufficient in settings where microbial contamination or biofilm formation is a clinical concern (Table 3).

### 3.2. Povidone-Iodine

Povidone-iodine (PI), commercially known as Betadine, is one of the most commonly used antiseptic agents [27] for intraoperative wound irrigation in orthopedic surgery [28]. Its clinical use began in the mid-20th century and remains prevalent due to its broad antimicrobial spectrum, affordability, and established safety profile when appropriately diluted [29,30]. The active ingredient, free iodine, exerts its antimicrobial effect by penetrating microbial cell walls and disrupting protein and nucleic acid structures through oxidative damage [28]. When applied intraoperatively, PI demonstrates bactericidal, fungicidal, virucidal, mycobactericidal, and sporicidal activity, making it effective across a wide range of pathogens including *Staphylococcus aureus*, *Streptococcus* spp., *Escherichia coli*, *Mycobacterium tuberculosis*, *Candida* spp., and enveloped viruses [7].

Povidone-iodine is typically administered intraoperatively as a dilute solution, with 0.35% concentration most commonly used during closure in total joint arthroplasty and spine surgery. It is delivered via bulb syringe, pulse lavage, or gravity-fed irrigation, with volumes ranging from 250 mL to 3 L depending on surgical complexity and the surface area of concern [7]. One of the most cited studies supporting its efficacy is a meta-analysis demonstrating a statistically significant reduction in surgical site infection (SSI) rates with intraoperative betadine irrigation compared to saline. Further studies in orthopedic subspecialties have shown reductions in both superficial and deep infections following PI use.

While its antiseptic efficacy is well-documented, concerns have emerged regarding its cytotoxic effects on host tissues [31]. In vitro studies have shown that PI, especially at higher concentrations, may impair the viability of osteoblasts, fibroblasts, chondrocytes, and other regenerative cells critical to wound healing and osseointegration [32]. Consequently, it is essential to use appropriate dilution and limit exposure time, with many protocols recommending thorough saline irrigation after PI application to reduce tissue toxicity. Its biofilm-disrupting properties are modest, and while it may assist in biofilm prevention, it does not reliably eradicate established biofilms [33].

Despite these limitations, PI remains a key component of many institutional and national guidelines for infection prevention during orthopedic surgery. Its cost-effectiveness, ease of use, and broad-spectrum coverage continue to make it a reliable tool, particularly in cases involving implants, revision procedures, or patients with elevated infection risk. Current best practices emphasize proper dilution, controlled exposure time, and consideration of tissue viability to maximize benefit while minimizing adverse effects.

### 3.3. Hydrogen Peroxide

Hydrogen peroxide (H_2_O_2_) is a traditional antiseptic with a long history in surgical wound management, first described for clinical use in the late 1800s [34]. Its antimicrobial activity is mediated through the generation of reactive oxygen species (ROS), particularly hydroxyl free radicals, which damage microbial membranes, proteins, and DNA [34]. Upon contact with catalase-positive organisms or host tissues, H_2_O_2_ decomposes into oxygen and water, creating an effervescent reaction that aids in the mechanical dislodgment of debris [35]. This dual mechanism—chemical and physical—makes hydrogen peroxide an effective agent for superficial wound cleaning and decontamination [22].

In orthopedic surgery, hydrogen peroxide is typically used at a concentration of 3%, applied in small volumes (usually 100–500 mL) via bulb syringe or soaked gauze [22]. It has demonstrated bactericidal and fungicidal properties, though its efficacy against spores and biofilm is limited. While hydrogen peroxide can reduce superficial bacterial load, it has not been shown to reliably penetrate or eradicate mature biofilm. Its antimicrobial effect is also transient, and the oxygen release during decomposition poses potential safety concerns [36].

One of the primary limitations of hydrogen peroxide is its cytotoxicity. Numerous in vitro studies have shown that it damages osteoblasts, chondrocytes, and fibroblasts, potentially impairing bone healing and tissue regeneration [7]. Additionally, its use near large vascular structures or within closed cavities carries a rare but serious risk of gas embolism. In implant-based surgeries, H_2_O_2_ can also contribute to corrosion of metallic components, particularly titanium and cobalt–chromium alloys [22,35]. For these reasons, its intraoperative use has significantly declined and is now largely restricted to select cases such as irrigation of grossly contaminated wounds or superficial debridement of devitalized tissue [37].

Despite its declining routine use, hydrogen peroxide remains a valuable adjunct in certain clinical scenarios when used judiciously. When incorporated into irrigation protocols, it is often followed by copious saline or antiseptic lavage to mitigate cytotoxic effects and remove residual oxygen bubbles [7]. However, in modern orthopedic practice, it is generally not recommended for use in deep wounds, periprosthetic spaces, or around neural and vascular structures due to its potential for tissue injury and embolic complications.

### 3.4. Acetic Acid

Acetic acid has a long-standing history as an antimicrobial agent, with documented use dating back over 6000 years [38]. As a weak organic acid, its antimicrobial mechanism is primarily driven by lowering local pH and generating anionic species that disrupt microbial cell walls and interfere with metabolic processes [39,40]. In recent decades, acetic acid has re-emerged in orthopedic surgery—particularly in revision and infected cases—due to its efficacy against biofilm-forming organisms [41].

Its resurgence was largely fueled by its ability to disrupt *Pseudomonas aeruginosa* biofilms, especially in burn care [42]. In vitro, acetic acid has demonstrated broad-spectrum antimicrobial activity, eradicating *P. aeruginosa*, *Proteus vulgaris*, *Acinetobacter baumannii*, *Streptococcus pyogenes*, *Staphylococcus aureus*, *S. epidermidis*, *Enterococcus faecalis*, and even *Mycobacterium tuberculosis* [43]. Concentrations as low as 0.16–0.31% have been shown to inhibit planktonic bacteria, with 3% producing complete eradication of multiple organisms within 30 min [40]. For biofilm eradication, 0.31% inhibited formation, while higher concentrations (e.g., 5%) eliminated over 96% of MRSA biofilm within 20 min. However, its efficacy is pH-dependent, with optimal performance near pH 4.76—an important consideration given that synovial fluid and blood have more alkaline pH values (~7.4), potentially limiting its activity in vivo [41].

In orthopedic procedures, acetic acid is most often delivered by bulb syringe or gravity-fed irrigation, using concentrations between 0.25% and 5%, and volumes typically ranging from 500 mL to 1 L. Its clinical application is largely restricted to infected wounds and debridement settings. In a 2017 study, Williams et al. incorporated a 20 min 0.19% acetic acid soak into the surgical protocol for periprosthetic joint infection (PJI) management during total knee arthroplasty, reporting safety and partial bactericidal efficacy [44]. Nevertheless, acetic acid was only bactericidal against 40% of isolates in that cohort, suggesting that while it may inhibit bacterial growth, complete eradication may require higher concentrations or longer exposure times [44].

Due to its demonstrated ability to prevent and disrupt biofilms, acetic acid remains a valuable adjunct in revision arthroplasty and chronic wound management. However, concerns over tissue irritation and the need for controlled exposure durations limit its widespread adoption in primary surgical cases.

### 3.5. Sodium Hypochlorite

Sodium hypochlorite, commonly referred to as Dakin’s solution, has been used in surgical care since World War I when it was developed by Henry Dakin and Alexis Carrel to treat infected wounds [45]. As a potent oxidizing agent, sodium hypochlorite exerts its antimicrobial effect by denaturing proteins and disrupting the integrity of microbial membranes and nucleic acids [34]. In dilute concentrations (typically 0.025–0.05%), it has broad-spectrum antimicrobial activity, effectively targeting Gram-positive and Gram-negative bacteria, fungi, and spores. However, its efficacy against biofilm is more limited and depends on concentration, exposure time, and the maturity of the biofilm [46].

In orthopedic surgery, sodium hypochlorite is usually delivered via gravity flow or bulb syringe in volumes ranging from 500 mL to 2 L [7]. Its application is most common in cases of gross contamination, infected wounds, or revision procedures where microbial burden is high. The solution is sometimes used as part of a sequential irrigation protocol, followed by saline or other antiseptic rinses to reduce tissue exposure. Dakin’s solution is particularly favored in wound care settings, such as open fractures or periprosthetic joint infections (PJIs), due to its ability to reduce microbial load quickly and cost-effectively [22,47].

Despite its antimicrobial potency, sodium hypochlorite is highly cytotoxic at concentrations above 0.05% [7]. Studies have demonstrated its damaging effects on osteoblasts, chondrocytes, fibroblasts, and muscle tissue, which can impair healing and increase the risk of soft tissue necrosis. Moreover, it has been shown to corrode metal implants, particularly titanium and cobalt–chromium alloys, making it a less favorable option in procedures involving internal fixation or arthroplasty [48,49]. Due to these risks, it is typically avoided in clean elective cases and used cautiously in the presence of hardware.

Although it offers reliable antimicrobial activity, sodium hypochlorite’s use is limited by its tissue toxicity and potential for implant corrosion [7]. It is best reserved for highly contaminated or infected surgical fields where bacterial eradication outweighs the risk of host tissue injury. When employed, it should be used in low concentrations, with controlled exposure time and subsequent saline irrigation to mitigate adverse effects.

### 3.6. Antibiotic Irrigation

The use of antibiotic irrigation in orthopedic surgery emerged in the mid-20th century as an adjunct to systemic prophylaxis, aiming to reduce surgical site infections by delivering high local concentrations of antibiotics directly to the wound bed. Commonly used agents include bacitracin, polymyxin B, gentamicin, vancomycin, and neomycin, either alone or in combination, typically diluted in normal saline and delivered intraoperatively via pulse lavage or bulb syringe in volumes ranging from 1 to 3 L [47]. The rationale is to suppress early contamination by targeting common skin flora and nosocomial pathogens before bacterial adherence and biofilm formation can occur.

Each antibiotic functions according to its specific mechanism of action: bacitracin inhibits cell wall synthesis in Gram-positive organisms, polymyxin disrupts the outer membrane of Gram-negative bacteria, vancomycin targets Gram-positive peptidoglycan layers, and gentamicin inhibits bacterial protein synthesis [7,28]. However, despite broad in vitro activity against planktonic bacteria, antibiotic irrigation is largely ineffective against established biofilm, spores, fungi, or mycobacteria [47]. Additionally, the brief exposure time during intraoperative lavage may be insufficient for meaningful bactericidal activity, particularly on implant surfaces or in avascular tissues [7].

The recent literature has cast doubt on the clinical benefit of routine antibiotic irrigation [50]. Multiple randomized controlled trials and meta-analyses have failed to demonstrate a significant reduction in infection rates compared to antiseptic or saline irrigation alone [51]. Moreover, concerns have grown regarding the development of antimicrobial resistance, allergic reactions, and anaphylaxis—especially with agents like bacitracin. The U.S. Food and Drug Administration (FDA) withdrew approval for bacitracin irrigation in 2020 due to safety concerns and lack of demonstrated efficacy [52]. Additionally, the cost-effectiveness of using large volumes of commercial antibiotic preparations intraoperatively remains debatable, particularly in the absence of strong supporting evidence.

Consequently, most contemporary guidelines discourage the routine use of antibiotic irrigation in primary arthroplasty and clean orthopedic procedures [7,23]. Instead, its use is typically reserved for specific high-risk scenarios, such as revision surgery for infection, gross contamination, or when culture-directed therapy is required intraoperatively. Even in these settings, its use should be guided by microbiologic data and balanced against potential risks to patient safety and public health.

### 3.7. Novel Citrate-Based Irrigation

Citrate-based irrigation solutions represent an innovative advancement in surgical infection control, developed to address the limitations of traditional antiseptics and antibiotics, particularly in the setting of biofilm-associated infections. One of the most well-studied formulations is XPERIENCE (Next Science LLC, Jacksonville, FL, USA), which contains citric acid, sodium citrate, and sodium lauryl sulfate [10]. This solution works through a multimodal mechanism: the citrate components chelate divalent metal ions (e.g., iron, calcium, magnesium) that are essential for the structural integrity of bacterial biofilms, effectively destabilizing the extracellular polymeric matrix. Meanwhile, sodium lauryl sulfate serves as a surfactant, reducing surface tension and facilitating the mechanical removal of bacteria and debris from the wound bed [53].

Unlike conventional antiseptics, citrate-based solutions are specifically designed for biofilm disruption and are applied intraoperatively via bulb syringe, pulse lavage, or gravity-fed systems [10]. Recommended volumes typically range from 500 mL to 2 L, depending on the procedure. A significant advantage of these formulations is their no-rinse design, providing up to five hours of antimicrobial protection post-application without needing to be rinsed [53]. This sustained activity is particularly beneficial in procedures involving implants, where biofilm prevention is critical in the early postoperative period.

In vitro studies have demonstrated robust antimicrobial activity across both Gram-positive and Gram-negative organisms [53], including *Staphylococcus aureus*, *Staphylococcus epidermidis*, *Escherichia coli*, and *Pseudomonas aeruginosa* [54]. The solution has been shown to reduce planktonic bacterial load by up to six logarithmic units and disrupt established biofilms with reductions of four to eight logs [55], depending on exposure time and strain [56]. Emerging evidence also suggests potential efficacy against fungal pathogens, although additional research is needed in this area [13].

Importantly, citrate-based solutions exhibit minimal cytotoxicity to human osteoblasts, fibroblasts, and chondrocytes, making them suitable for use around bone, cartilage, and soft tissues [10]. Clinical studies in joint arthroplasty have reported decreased postoperative swelling, improved early range of motion, and reduced opioid consumption in patients treated with XPERIENCE™ compared to povidone-iodine [13,56,57]. A retrospective study by Singer et al. [12] of 524 primary TKAs performed at an ambulatory surgery center revealed a PJI rate of 0.19%, highlighting its relative efficacy in preventing infections. Another review by Williams et al. of 423 primary total joint arthroplasties (knee, hip, and shoulder) revealed a 0% PJI rate, further highlighting its relative efficacy in preventing prosthetic joint infections [57]. Of note, a pilot study of 54 patients undergoing primary TKA revealed citrate-based irrigation use was associated with decreased post-operative swelling and improved patient comfort overall [58]. Furthermore, early infection outcomes—though not always statistically significant—consistently trend toward a reduced rates of surgical site infection and reoperation.

While large-scale randomized controlled trials are still ongoing, the accumulating basic science and clinical data support citrate-based irrigation as a promising alternative in both primary and revision orthopedic procedures. Its favorable safety profile, sustained antimicrobial activity, and strong biofilm-targeting mechanism distinguish it from other irrigation modalities, suggesting a potential future role in standard perioperative protocols for infection prevention.

## 4. Current/Future Clinical Trials

A growing body of clinical research is underway to more clearly define the role of citrate-based intraoperative irrigation solutions in orthopedic surgery [11] particularly in the prevention of periprosthetic joint infection (PJI) (Table 3). Among the most significant ongoing trials is a multicenter, double-blinded randomized controlled trial led by the Ottawa Hospital (NCT05543941), which compares a citrate-based solution to dilute povidone-iodine in patients undergoing total hip and knee arthroplasty or hip resurfacing [59]. The study’s primary endpoint is the rate of PJI within 90 days, with secondary outcomes including superficial surgical site infections, one-year PJI rates, and subgroup analysis of high-risk populations, including those with diabetes, inflammatory arthritis, and chronic kidney disease. Complementing this effort is a prospective randomized study at Northwell Health (NCT05519007), enrolling 936 patients at high risk for PJI undergoing total hip arthroplasty [60]. This trial compares citrate-based irrigation to normal saline, with deep postoperative infection at three months as the primary endpoint. These studies are expected to provide definitive data on the clinical utility of this irrigation strategy in large-joint arthroplasty.

Several additional trials are in development to extend the evaluation of this solution into other orthopedic domains. A shoulder arthroplasty study will assess its efficacy against *Cutibacterium acnes* when compared to hydrogen peroxide and povidone-iodine [61]. Other upcoming trials aim to evaluate its role in reducing postoperative swelling in total hip arthroplasty and hip surgery overall (including resurfacing) (Table 4).

Together, these investigations represent a comprehensive effort to assess whether intraoperative use of citrate-based irrigation can effectively reduce bacterial colonization, prevent biofilm formation, and minimize postoperative complications. While early pilot studies suggest favorable outcomes in terms of swelling, pain, and infection control, these ongoing and future trials will be critical in establishing broader clinical adoption and guiding standardized irrigation protocols across orthopedic surgery. Furthermore, it is encouraged that future studies consider combinations of irrigation solutions, especially in high-risk procedures (patients with multiple co-morbidities, revision surgery, and/or trauma/infection cases).

## 5. Conclusions

Intraoperative irrigation remains a cornerstone of infection prevention in orthopedic surgery, yet many traditional solutions—such as saline, povidone-iodine, hydrogen peroxide, and hypochlorite—are limited by cytotoxicity, lack of sustained antimicrobial effect, or inadequate biofilm disruption. Among the agents reviewed, citrate-based irrigation solutions offer a superior profile by combining effective microbial eradication with biofilm breakdown, prolonged antimicrobial activity, and minimal harm to host tissues. Preliminary clinical studies have demonstrated reduced infection rates, improved early postoperative recovery, and favorable outcomes in both primary and high-risk arthroplasty settings. As ongoing randomized trials continue to validate these early findings, citrate-based solutions are poised to redefine the standard of care for intraoperative wound irrigation in orthopedic procedures.

## Figures and Tables

**Table 2:** Advantages/disadvantages of common intraoperative irrigation solutions.

Irrigation Solution	Advantages	Disadvantages
Normal Saline	Biocompatible; inexpensive; widely available; safe for host tissues	No antimicrobial or biofilm activity; mechanical cleansing only
Povidone-Iodine	Broad-spectrum antimicrobial; inexpensive; effective at appropriate dilution	High cytotoxicity if not diluted; short antimicrobial duration; requires rinse
Hydrogen Peroxide	Bactericidal; mechanical effervescence aids debridement; low cost	High cytotoxicity; transient effect; gas embolism risk; implant corrosion
Acetic Acid	Effective against Pseudomonas and biofilm; low cost; broad antimicrobial activity	High cytotoxicity; pH-dependent efficacy; limited routine use
Sodium Hypochlorite	Broad-spectrum antimicrobial; cost-effective; effective in gross contamination	High cytotoxicity; implant corrosion; limited use in clean surgeries
Antibiotic Irrigation	Targeted antimicrobial activity; tailored to specific pathogens; widely used historically	Limited biofilm activity; resistance risk; hypersensitivity; preparation complexity
Citrate-Based Solution	Strong biofilm disruption; prolonged antimicrobial activity; low cytotoxicity; no rinse required	Higher cost; emerging clinical evidence still maturing; limited fungal/spore data

**Table 3:** Usability, safety, and practical considerations of orthopedic irrigation solutions.

Irrigation Solution	Ease of Use (One-Time Application)	Cytotoxicity to Host Cells	Antimicrobial Duration	Residual Protection	Resistance Risk	Approximate Cost (USD)
Normal Saline	Very easy	None	None	None	None	<1 $/L
Povidone-Iodine (Betadine)	Moderate (requires dilution)	High	Very short (<10 min)	None	None	~170 $/L
Hydrogen Peroxide	Easy	High	Very short (<10 min)	None	None	~1–3 $/L
Acetic Acid	Moderate	High	Very short (<10 min)	None	None	~450 $/L
Sodium Hypochlorite	Moderate	High	Short	Minimal	None	~1–3 $/L
Antibiotic Irrigation	Complex (requires preparation)	Low	Short	None	High	~20–50 $/L
Citrate-Based Solution	Very easy (ready to use, no rinse)	Low	Long (up to 5 h)	Yes	None	~200 $/L

**Table 4:** Ongoing/future clinical trials planned for novel citrate-based irrigation.

Trial Name/Identifier	Irrigation Type	Surgical Context	Number Patients	Primary Outcome	Lead Institution
**NCT05519007**	Citrate-Based Solution vs. Saline	Total Hip Arthroplasty	936	Reduction in postoperative pain and opioid use	Northwell
**NCT05543941**	Citrate-Based Solution vs. Povidone-Iodine	Total Knee Arthroplasty, Total Hip Arthroplasty, Hip Resurfacing	7600	Periprosthetic joint infection rate < 90 days post-surgery, superficial wound infections, patient-reported functional outcomes at 1 year	Ottawa Hospital
**NCT06831422**	Citrate-Based Solution vs. 3% Hydrogen Peroxide vs. 10% Povidone-Iodine	Primary Shoulder Arthroplasty	150	Incidence of *C. acnes* up to 18 days, perioperative complications and re-operations up to 1 year	Henry Ford
**NCT06126614**	Povidone-Iodine vs. Vancomycin vs. Saline	Total Joint Arthroplasty (TKA/THA/TSA)	21,006	Reoperations due to infection at 1 year, surgical site infections requiring antibiotics and treatment up to 1 year	McMaster University
**Spine Infection (Planned)**	Citrate-Based Solution vs. Saline	1–2 Level Posterior Lumbar Laminectomy/Fusion	~50	Surgical site infection rate, re-admissions, re-operations	Maimonides Medical Center
